# Continuous data capture of gait and mobility metrics using wearable devices for postoperative monitoring in common elective orthopaedic procedures of the hip, knee, and spine: a scoping review

**DOI:** 10.1186/s13018-023-04303-5

**Published:** 2023-10-31

**Authors:** Pragadesh Natarajan, R. Dineth Fonseka, Monish Movin Maharaj, Lianne Koinis, Ralph Jasper Mobbs

**Affiliations:** 1 Faculty of Medicine, University of New South Wales, Sydney, Australia; 2 NeuroSpine Surgery Research Group (NSURG), Sydney, Australia; 3 Wearables and Gait Analysis Research Group (WAGAR), Sydney, Australia; 4 Department of Neurosurgery, Prince of Wales Hospital, Sydney, Australia

**Keywords:** Wearable monitoring, Post-operative recovery, Orthopaedic surgery, Hip, Knee, Spine

## Abstract

**Background:**

Surgical procedures involving the hip, knee, or spine represent a majority of orthopaedic procedures performed electively in the health care system. Postoperative care is a key aspect of surgery and mobilisation without injury is the primary objective. Recent advances in wearable technologies allow objective evaluation of walking metrics to inform and guide postoperative care following orthopaedic surgery.

**Purpose:**

The aim of this scoping review is to explore current applications of wearable devices, objective data capture and gait analysis in monitoring postoperative recovery following commonly performed elective orthopaedic procedures of the hip, knee and spine.

**Methods:**

A search against pre-defined criteria was performed on the following scientific databases from date of inception to February 28th, 2021: Medline (via OvidSP), Embase (via OvidSP) and Cochrane Library (via CENTRAL). Data were collected according to a predetermined checklist including study participants, surgery, wearable device (model), sensor location, and monitoring parameters such as mobility metrics, monitoring timepoints and monitoring duration for each study included in our review. Quality was assessed independently using the Newcastle Ottawa Scale (NOS).

**Conclusions:**

To our knowledge, this is the first review of wearable monitoring (of postoperative recovery) following hip, knee and spine surgery. Patients undergoing elective orthopaedic procedures may benefit from wearable monitoring of their walking health and mobility metrics.

## Background

Musculoskeletal conditions account for more disability and more costs to the United States health care system than any other condition [1]. When conservative treatment options fail, these diseases may be managed surgically. Over 200 000 total hip arthroplasty and 600 000 total knee arthroplasty procedures are performed per year in the USA [2, 3]. Similar high volumes of 3.1 million total hip arthroplasties and 2.5 million total knee arthroplasties are performed every year in Europe, as well as over 95,000 joint replacements performed every year in Australia [4–6]. Total Medicare reimbursements for lumbar surgery alone in the USA exceeds $1 billion per year [7]. Together, surgical procedures involving the hip, knee, or spine represent a majority of orthopaedic procedures performed electively in the health care system and form a significant proportion of all surgical procedures performed by a typical hospital by both sheer case numbers and expenses.

Postoperative care is a key aspect of surgery and involves facilitating safe recovery. In the context of orthopaedic procedures especially, mobilisation without injury is the primary objective during postoperative rehabilitation [8]. Other objectives may include the early detection of any postoperative complications. Typically, patients receive immediate postoperative care as an inpatient until discharge followed by outpatient clinic visits of diminishing frequency [8]. Further, assessment of postoperative outcomes may be obtained at arbitrarily fixed timepoints, via patient reported outcome measures (PROM) such as the Owestry Disability Index, Oxford Knee Score and the Hip disability and Osteoarthritis Outcome Score. Although these questionnaire-based clinical tools offer meaningful insight into a patient’s functional outcomes such as extent of disability and impact on activities of daily living, they are limited by subjectivity due to patients’ reporting bias and mode of administration. [9, 10]

Walking is an essential activity of daily living, and is directly related to the function and health of mechanical, musculoskeletal and neurological systems [11]. Commonly dubbed as “ the sixth vital sign”, walking metrics such as gait velocity and step count are important indicators of not only general health status but also decline and recovery [12]. Moreover, these walking metrics provide an objective alternate measure of functional outcomes and disability to the inherently subjective patient reported outcome measures.

Recent advances in wearable technologies allow objective evaluation of these walking metrics to inform and guide postoperative care following elective orthopaedic surgery. ‘Wearable devices’ (***wearables***) containing various microelectromechanical sensors (MEMS) such as accelerometers and/or gyroscopes have recently emerged as a method of objectively measuring walking metrics. These devices can accurately capture a range of metrics including simple mobility metrics such as step count and physical activity levels to complex walking parameters, such as gait velocity, cadence, and stride length [13]. Advantageously, they are small, cheap, and marry the convenience of at-home postoperative monitoring with accuracy and objectivity. They can be worn at a single point on the body or multiple points, can function on their own, or be incorporated into various devices, such as watches, phones, jewellery, pendants, or insoles [14]. Most notably, wearable monitoring offers objective and continuous data capture of these ‘walking metrics’ to monitor patient recovery. Unlike PROMS, which offer a “snapshot” into a patient’s functional status at a particular point in their ongoing recovery, wearable devices enable continuous data capture of their mobility data to more holistically detail patients’ recovery.

The objective of this scoping review is to explore current applications of wearable devices, objective data capture and gait analysis in monitoring postoperative recovery following elective orthopaedic procedures. Procedures of the hip, knee and spine are amongst the most common. Therefore, eight commonly undertaken elective orthopaedic procedures were considered for inclusion: total hip replacement, total knee replacement, arthroscopic anterior cruciate ligament reconstruction, arthroscopic meniscal repair of the knee, arthroscopic partial meniscectomy of the knee, lumbar spine decompression and lumbar spine fusion.

## Methods

### Eligibility criteria

The focus of this scoping review was on published original articles written in English and published between 1980 and August 2021, including all study designs such as case reports, short series, cohort studies, randomised trials, or other study designs. The PRISMA statement guidelines were followed in identifying, screening, and selecting studies for inclusion, and extracting data.

### Inclusion criteria

1. Articles involving wearable devices for the purpose of continuous and objective monitoring of postoperative recovery.
2. The wearable device is capable of measuring gait or mobility metrics.
3. Postoperative monitoring after the following orthopaedic procedures:
  - total hip replacement,
  - total knee replacement,
  - arthroscopic anterior cruciate ligament reconstruction,
  - arthroscopic meniscal repair of the knee,
  - arthroscopic partial meniscectomy of the knee,
  - lumbar spine decompression
  - lumbar spine fusion
4. Articles written in English.
5. Articles published between 1980 – August 2021.

### Exclusion criteria

1. Wearable technology studies involving non-mobility or gait data capture.
2. Studies assessing patient function during a single walking bout (non-continuous)
3. Studies involving robotic ‘feedback’ wearables, exoskeletons or smartphones.
4. Studies of artificial intelligence algorithms or predictive modelling of patient outcomes
5. Systematic Reviews
6. Conference Abstracts

### Search strategy

Relevant studies were identified through a systematic search for published papers in the following scientific databases from date of inception to February 28th, 2021: Medline (via OvidSP), Embase (via OvidSP) and Cochrane Library (via CENTRAL). The search ‘concepts’ were *wearable (gait-tracking) devices* and *elective orthopaedic procedures* (see Table 1).

**Table 1:** Search Strategy

Wearable Device	Patient Population						
	Total knee replacement	Total hip replacement	Arthroscopic anterior cruciate ligament reconstruction	Arthroscopic meniscal repair of the knee	Arthroscopic partial meniscectomy of the knee	Lumbar spine decompression	Lumbar spine fusion
1. exp Wearable Electronic Devices/ 2. exp Fitness Trackers/ 3. (wearable* OR wearable device* OR acceleromet* OR gyroscope OR magnetometer OR inertial measurement unit OR IMU OR sensor OR activity tracker).mp 4. 1–3 OR/	5. exp Arthroplasty, Replacement, Knee/ 6. exp osteoarthritis/ 7. 5 AND 6	8. exp Arthroplasty, Replacement, Hip/ 9. exp osteoarthritis/ 10. 8 AND 9	11. exp Arthroscopy/ or arthroscopic.mp 12. exp Anterior Cruciate Ligament Reconstruction/ 13. ACL reconstruction.mp 14. ACLR.mp 15. 12–14 OR/ 16. 15 AND 11	17. exp Arthroscopy/ or arthroscopic.mp 18. meniscus repair.mp 19. meniscal repair.mp 20. meniscal surgery.mp 21. 18–20 OR/ 22. 21 AND 17	23. exp Arthroscopy/ or arthroscopic.mp 24. exp Meniscectomy/ 25. menisc*.mp 26. 24 OR 25 27. 26 AND 23	28. exp Decompression, Surgical/ or lumbar decompression.mp 29. spinal decompression.mp 30. lumbar spinal decompression.mp 31. 18–30 OR/	32. exp Spinal Fusion/ 33. exp Lumbar Vertebrae/ or lumbar.mp 34. exp Intervertebral Disc Degeneration/ or degenerative dis*.mp 35. 32–34 AND/ 36. 7 OR 10 OR 16 OR 22 OR 27 OR 31 OR 35 37. 36 AND 4

### Study selection

The literature search was completed by two authors (PN and RDF). Titles and abstracts of all studies identified were screened for relevance. Studies which were not relevant based on the title and abstract screen were excluded from the review. The full text of the record was reviewed if relevance was uncertain, and third reviewer consulted (RJM) if necessary until consensus agreement was reached regarding inclusion/exclusion. The full text of all selected relevant records was reviewed, and eligibility was determined using the eligibility criteria defined above. The quality of each included record was assessed by two authors (PN and RDF), and relevant information extracted.

### Data collection

Following the selection of articles, data was collated by two reviewers (PN and RDF). Data were collected according to a predetermined checklist including: study participants, surgery, wearable device (model), sensor location, and monitoring parameters such as mobility metrics, monitoring timepoints and monitoring duration for each study included in our review. Each study was also appraised independently for bias using the Newcastle Ottawa Scale by two reviewers (PN and RDF) and a third senior reviewer consulted for discrepancies (MM) [15]. Quality assessments from the Newcastle–Ottawa scale was converted to summary categories of good, fair, and poor quality according to the Agency for Healthcare Research and Quality (AHRQ) standards.

## Results

**Fig. 1:**
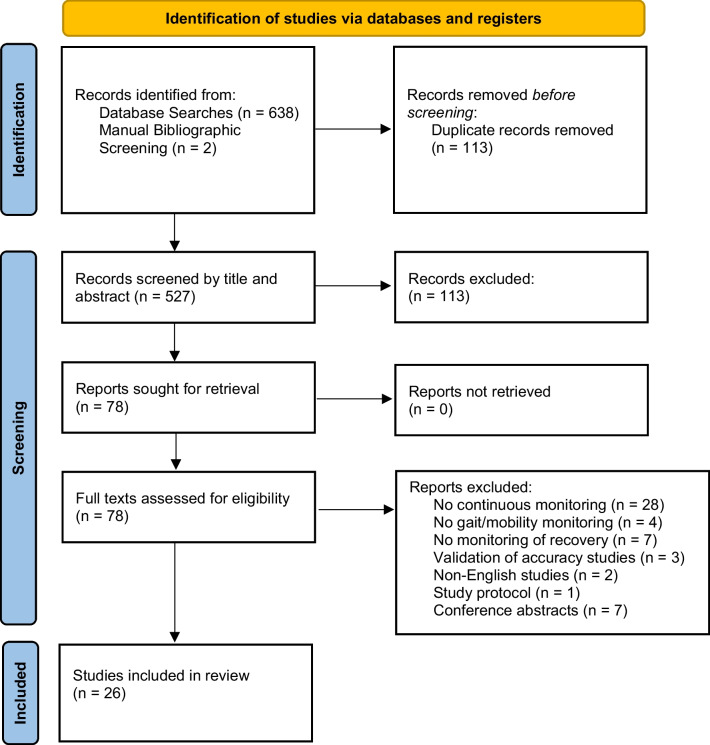
Flow diagram for study selection and searches of databases and registers

Following database searches for each orthopaedic procedure we identified a total of 640 relevant records (see Table 2). After removal of duplicates, 527 studies remained. Four hundred forty-nine references were excluded on title and abstract screen. A further 53 articles were excluded upon full-text review, leaving a final 26 studies to be included in qualitative synthesis. A flowchart of this process is shown in Fig. 1.

**Table 2:** Search Results

	Total knee replacement	Total hip replacement	Arthroscopic anterior cruciate ligament reconstruction	Arthroscopic meniscal repair of the knee	Arthroscopic partial meniscectomy of the knee	Lumbar spine decompression	Lumbar spine fusion	Total
**Medline**	92	30	75	0	7	35	5	244
**Embase**	109	22	23	6	27	157	4	348
**Cochrane Library (CENTRAL)**	9	27	2	0	0	5	3	46
**Bibliographies**								2
**Total Records Identified**								640
Duplicates excluded								113
**Records screened title and abstracts**								527
Records excluded								449
**Full texts assessed for eligibility**								**78**
Records excluded								**52**
**Studies included in qualitative synthesis**								**26**

The 26 included studies comprised the procedures of ACL reconstruction (1 study) [16], Lumbar Decompression (2 studies) [17, 17], Lumbar Fusion (5 studies) [18–22], TKR (9 studies) [23–31] and THR procedures (9 studies) [23–31] as seen in Table 3. No studies related to arthroscopic partial meniscectomy and meniscal repairs of the knee were retrieved from the literature search. Sample sizes ranged from 12 participants [32] to as high as 242 participants [33]. A detailed summary of these studies is included in Table 4. Due differences in study design, wearable monitoring protocols and surgical cohorts between studies it was not possible to meta-analyse findings from included studies.

**Table 3:** Wearable monitoring of postoperative recovery following orthopaedic surgery

Procedure	Studies
Arthroscopic anterior cruciate ligament reconstruction	1
Arthroscopic meniscal repair of the knee	0
Arthroscopic partial meniscectomy of the knee	0
Lumbar spine decompression	2
Lumbar spine fusion	5
Total hip replacement	9
Total knee replacement	8

**Table 4:** Summary of included studies

Authors	Population		Wearable		Monitoring		
	Participants (N)	Surgery	Device	Location	Mobility Metrics	Timepoints	Duration
[32]	End-stage knee osteoarthritis n = 33	Total knee arthroplasty	ActivPAL (PAL Technologies Ltd., Glasgow, United Kingdom)	Upper thigh	Sedentary time, Standing time, Upright time, Stepping time, Step count	Preoperatively, 6 months Postoperatively, 12 months postoperatively	7–8 days
[24]	Osteoarthritis N = 53	Total knee arthroplasty	1 Step activity monitor and 2 ADL monitors (McRoberts)	RIght ankle (step) Waist belt, anterior thigh (ADL)	Gait cycles, Walking time Resting time	preoperatively, 2 months Postoperatively, 6 months postoperatively, 12 months postoperatively	7 days
[25]	End-stage gonarthrosis N = 36	Primary total knee arthroplasty	ActiCal (Philips Respironics, Bend, OR, USA)	Waist	Sedentary awake time, Light physical activity time, Moderate-vigorous physical activity time, Daily energy expenditure, Step count	Preoperatively, 6 months Postoperatively	at least 5 days (10 + hours / day)
[35]	Hip osteoarthritis N = 60	Total hip arthroplasty	ActivPAL (PAL Technologies Ltd., Glasgow, United Kingdom)	Anterior thigh (non-operative)	Upright time per 24 h, Walking time, Number of steps, Number of upright events/day	First postoperative week	6–7 days (24 h/day)
[26]	End-stage knee osteoarthritis N = 89	Total knee arthroplasty	ActiGraph GT3X +	Waist (operative)	Sedentary time, Light intensity activity time, Moderate intensity activity time, Vigorous intensity activity time, Step count	Preoperatively, 6 weeks postoperatively, 6 months postoperatively	7 days (10 + hours/day)
[27]	Knee osteoarthritis N = 73	Total knee arthroplasty	ActivPAL (PAL Technologies Ltd., Glasgow, United Kingdom)	Anterior thigh (operative)	Sitting time, Standing time, Stepping time, Step count	Preoperatively, 6 weeks Postoperatively, 6 months postoperatively	7 days (24 h / day)
[19]	Lumbar Spine N = 216	Lumbar decompression, discectomy and/or fusion	ActivPAL3 (PAL Technologies Ltd., Glasgow, United Kingdom)	Anterior thigh	Step count, Stepping time, Standing time, Sedentary time, Number of walking episodes > 1, 5, and 10 min long	First postoperative week	8 days (8am on surgery morning - 8am on eighth day) 24 h a day
[36]	Hip or knee osteoarthritis (N = 57)	Total hip arthroplasty or total knee arthroplasty	ActiGraph GT1M (ActiGraph LLC, Fort Walton Beach, FL, USA)	Waist	Activity counts, Proportion of time engaged in sedentary activity (activity intensity)	Preoperatively, 6 months postoperatively	Minimum of 4 of the 7 days (10 + hours/day)
[20]	Lumbar spinal stenosis (N = 42), lumbar spondylolisthesis (N = 13), degenerative lumbar scoliosis (N = 5)	Lumbar decompression (n = 26) or lumbar fusion (n = 34)	Actigraph® Micro-Motion logger (Ambulatory Monitors Inc., Ardsley, NY, USA)	Wrist (non-dominant)	Activity counts	Preoperatively, 1 month postoperatively, 3 months postoperatively, 6 months postoperatively, 12 months postoperatively	7 days (8AM to 6PM)
[37]	Hip or knee osteoarthritis N = 24	Total hip arthroplasty or total knee arthroplasty	ActiGraph wGT3X-BT accelerometer (v1.9.2, ActiGraph, LLC, Fort Walton Beach, FL, USA)	Waist (dominant)	Total time in sedentary bouts, Length of sedentary bouts, Step count, Total time in moderate-vigorous physical activity	Preoperatively Postoperatively (mean time between timepoints = 11.8 months)	5 – 7 days, 16 h per day
[39]	Hip or Knee osteoarthritis N = 146	Total hip arthroplasty or total knee arthroplasty	Nokia Go (Nokia Europe, Issyles-Moulineaux, France)	Wrist	Step count	Preoperatively, 3 months postoperatively	5–7 days. further detail not specified
[27]	Knee Osteoarthritis N = 51	Total knee replacement	Fitbit Flex-2 wristband	Wrist	Time in moderate-vigorous physical activity, Time in purposeful activity, Step count, Sedentary time	Preoperatively, 13 weeks postoperatively, 26 weeks postoperatively, 39 weeks postoperatively	7 days, 24 h/day
[33]	Advanced hip osteoarthritis N = 12	Total hip arthroplasty	RT3 accelerometer (StayHealthy, Inc., Monrovia, CA)	Waist	Energy expenditure	One month preoperatively Six months postoperatively	21 days
[29]	Knee osteoarthritis N = 66	Primary total knee arthroplasty	Lifecorder EX (Suzuken Co. Ltd, Nagoya, Japan)	Waist	Light physical activity time, Moderate-vigorous physical activity time, Total physical activity time, Step count	One month preoperatively Six months postoperatively	7 days
[29]	Hip osteoarthritis N = 153	Total hip arthroplasty	Lifecorder EX (Suzuken Co. Ltd, Nagoya, Japan)	Waist	Step count, Moderate-vigorous physical activity time	Preoperatively, One year postoperatively, Three years postoperatively	7 days
[34]	Knee or hip osteoarthritis N = 242 (96 received remote monitoring)	Total hip arthroplasty or total knee arthroplasty	Withings physical activity monitor (Withings, France)	Wrist	Step count	Two weeks postoperatively six weeks postoperatively	Study duration (8 weeks)
[17]	Lumbar spinal stenosis N = 13	Lumbar decompression	MiBand2 (Xiaomi, China) or the patient’s own device, according to personal preference	Wrist	Step count	Preoperatively Postoperatively (mean time between timepoints = 92 days)	Throughout duration of study period (3 months)
[21]	Low back pain, radiculopathy or neurogenic claudication N = 30	Anterior lumbar interbody fusion (n = 7), laminectomy (n = 13), posterior lumbar interbody fusion (n = 2), or discectomy (n = 6)	Fitbit zip accelerometer (Fitbit Inc., San Francisco, California, United States)	Wrist	Step count, Distance travelled, Calorie expenditure	Preoperatively, One month postoperatively, Two months postoperatively, Three months postoperatively	Throughout duration of study period (3 months)
[30]	Osteoarthritis N = 25	Primary total knee arthroplasty	iPhone	Pocket	Step count	Preoperatively to 3 months postoperatively	Worn throughout the duration of the study period. (3 months)
[20]	Adult spinal deformity or degenerative spinal disease N = 32	Lumbar surgery	Fitbit Flex (Fitbit Inc., San Francisco, California, United States)	Wrist	Step count, Maximum hourly steps, Time spent sedentary, Time spent lightly active, Time spent fairly active, Time spent very active	One month preoperatively to at least six months postoperatively	Worn throughout the duration of the study period, except for the first postoperative month (6 months)
[16]	Acute anterior cruciate ligament tear N = 60	Dynamic intraligamentary stabilization or anterior cruciate ligament reconstruction	StepWatch Activity Monitor (Modus Health, Washington DC, USA)	Unspecified	Step count	First six weeks postoperatively	Throughout Duration of First six postoperative weeks
[18]	Lumbar spinal stenosis N = 38	Surgical treatment of lumbar spinal stenosis	ActiGraph GT3X accelerometer (ActiGraph, LLC; Ft. Walton Beach, FL)	Hip	Moderate-vigorous activity minutes, Sedentary range activity, Light range activity, Moderate range activity, Vigorous range activity	One week preoperatively, Six months postoperatively	7 days
[23]	Disorders of the cervical or lumbar spine N = 30	Lumbar discectomy, lumbar decompression, lumbar fusion, anterior cervical discectomy and fusion, and, posterior cervical decompression and fusion	Mi Band (Xiaomi, Mountain View, CA (USA)	Wrist	Step count	preoperatively, 1, 2, 4, 8, 12, 26, and 52 weeks postoperatively	7 days
[31]	Knee osteoarthritis N = 92	Total knee arthroplasty	ES-500 Pedometer (YAMASA, Japan)	Unspecified	Step count	Preoperatively until 6 months postoperatively	Throughout the duration of this time period, (6 months)
[40]	Hip osteoarthritis, or avascular necrosis of the femoral head, or inflammatory arthritis of the hip N = 51	Primary total hip arthroplasty	GeneActiv (Cambridge, United Kingdom)	Wrist (non-dominant)	Number of sedentary bouts per day, Mean sedentary bout length, Dispersion of sedentary bouts throughout the day, Time spent in light physical activity, Time spent in moderate physical activity, Time spent in vigorous physical activity	Preoperatively, 6 weeks, 3 and 6 months postoperatively	7 days, 24 h per day
[41]	End-stage hip osteoarthritis N = 60	Total hip arthroplasty	Activity Monitor, consisting of four ADXL202 devices (Analog Devices, Breda, The Netherlands, adapted by Temec Instruments, Kerkrade, The Netherlands)	Thigh (both sides), trunk, and lower arm (both sides)	TIme spent walking, Number of walking periods, Duration of walking periods, Body motility during walking, stride frequency, tTme spent sitting, Duration of sitting periods, Number of chair risings, Duration of chair rising movement	preoperatively 6 months postoperatively	Unspecified

Commonly employed devices for continuous wearable monitoring were ActivPAL (PAL Technologies Ltd., Glasgow, United Kingdom) [18, 26, 31, 34], ActiGraph GT1M (ActiGraph LLC, Fort Walton Beach, FL, USA) [17, 19, 36, 37], Lifecorder EX (Suzuken Co. Ltd, Nagoya, Japan) [28, 35] and MiBand2 (Xiaomi, China) [17, 22]. Some studies also employed consumer fitness wearable devices such as Fitbit (Fitbit Inc., San Francisco, California, United States) [20, 21, 27] and Withings (Withings Inc, France) [33]. Other studies permitted use of patients’ own device and/or involved the use of their smartphone’s step counter functionality. [17, 29]

Most common sensor placement locations employed included wrist [17, 20–22, 27, 33, 34, 36, 37], waist [19, 23–25, 28, 32, 35–37] and thigh [18, 23, 26, 31, 34]. Although majority involved single-point wearables, a select few studies employed more than one wearable device [23, 38]. Captured data typically comprised of physical activity intensities (for example light, moderate or vigorous) and physical activity durations (for example sitting, standing, with few studies also collecting basic spatial and temporal gait metrics such as step count [16–18, 20–22, 25–30, 33–37], gait cycles [23] and stride frequency [38]. Some studies additionally collected caloric/energy expenditures [20, 24, 32]. Studies typically monitored patient mobility at specific timepoints of recovery such as several weeks, 3 months, and 6 months postoperatively (as seen in Table 4). However few studies monitored the entire recovery period from operative timepoints to 6 months postoperatively [16, 17, 20, 21, 30, 33], with majority monitoring “recovery windows” at perioperative and/or post-recovery timepoints.

In terms of quality of included studies, most were of good quality according to the AHRQ standards. Of the 26 included studies, 22 studies [16, 17, 17–20, 22–31, 33, 34, 36, 36–38] were of good quality, with 3 studies [21, 32, 35] of fair quality and 1 study of poor quality, as seen in Table 5. However, 7 had short follow-up durations (< 3 months) [17, 20, 29, 33, 34, 36, 37] with 12 studies reporting follow-up data on less than 80% of recruited participants. [16–19, 21, 23, 25, 26, 32, 36, 37, 37]

**Table 5:** Quality assessment of included studies using Newcastle Ottawa Scale for cohort studies

Study	Selection				Comparability	Outcome			Quality
	Representativeness of exposed	Selection of non-exposed	Ascertainment of exposure	Assessment of outcomes at study commencement	Comparability of cohorts	Assessment of outcome	Follow-up duration long enough (> 3 m)	Follow up of cohort is adequate (> 80%)	
[32]	*	0	*	*	**	*	*	*	Good
[24]	*	0	*	*	**	*	*	0	Good
[25]	*	0	*	*	**	*	*	*	Good
[35]	*	*	*	0	**	*	0	*	Good
[26]	*	0	*	*	**	*	*	0	Good
[27]	*	0	*	*	*	*	*	0	Good
[19]	*	0	*	*	**	*	*	0	Good
[36]	*	0	*	*	**	*	*	0	Good
[20]	*	0	*	*	**	*	*	0	Good
[37]	*	*	*	0	**	*	*	0	Good
[39]	*	0	*	*	**	*	0	*	Good
[27]	*	*	*	*	**	*	*	*	Good
[33]	0	0	*	*	**	*	*	0	Fair
[29]	*	*	*	*	**	*	*	*	Good
[30]	*	0	*	0	**	*	*	*	Fair
[34]	*	*	*	0	**	*	0	*	Good
[21]	*	0	*	*	**	*	0	*	Good
[17]	*	0	*	*	**	*	0	*	Good
[30]	*	0	*	*	**	*	0	*	Good
[22]	0	0	*	*	**	*	*	0	Fair
[16]	*	*	*	*	*	*	*	0	Good
[18]	*	0	*	*	**	*	*	0	Good
[23]	*	0	*	*	**	*	*	*	Good
[31]	*	0	*	*	**	*	*	*	Good
[40]	*	0	*	*	**	*	0	0	Poor
[41]	*	*	*	*	*	*	*	*	Good

## Discussion

The findings of the present review demonstrate thus far supportive data for clinical applications of wearable monitoring of patient recovery following common elective orthopaedic surgeries. However, higher quality evidence with large-volume studies is needed, with applications following some surgeries such as arthroscopic meniscal repair of the knee, and arthroscopic partial meniscectomy of the knee, yet to be validated. Moreover, current studies are limited to basic mobility metrics such as step count and activity profiles. Future studies may incorporate other quantitative gait metrics (beyond step count) such as gait velocity, step or stride length, gait asymmetry and gait variability. Most studies are single-centre clinical series with small to moderate sample sizes. Notably prevalent are patient compliance issues, with included studies typically reporting follow-up data on less than 80% of recruited participants, as seen in Table 5.

Included studies demonstrated a wide variety of uses and benefits for wearable monitoring. Benefits of wearable devices in facilitating remote patient monitoring has been reported by numerous authors with Ramkumar et al. [29] suggesting the possibility of real-time collection of other data such as range of motion, patient reported outcome measures, opioid consumption, and home exercise compliance. Gamification and remote monitoring was reported as a means of improving recovery outcomes following knee and hip arthroplasty in Mehta et al. (2020)’s randomised clinical trial [33]. Although wearable monitoring was found to offer no direct effect as an intervention (in improving mobility levels), the rate of rehospitalisation was found to be significantly reduced (3.4% versus 12.2%, *p* = 0.01) suggesting overall benefits to recovery outcomes. By contrast, a multi-model wearable monitoring program coupled with physical therapy counselling by Li et al. [27] resulted in mean improvements in (moderate-vigorous) physical activity levels of 13.1 min per day (95% CI 1.6 to 24.5). Despite these discrepancies in which outcomes are improved, it is likely wearable monitoring offers some sort of benefit to postoperative care and recovery.

Other uses of wearable monitoring that are yet to be explored in larger orthopaedic surgery cohorts includes the screening and early detection of complications in the peri- and postoperative period. The detection of recurrent disc herniation following microdiscectomy has previously been detailed by Mobbs et al.’s case report in 2018 [39], suggesting such wearable monitoring for postoperative complications may be clinically feasible.

Another application of wearable monitoring may be the tracking of postoperative recovery against “normalised” trajectories to guide mobility interventions. For example, Carmichael et al. (2019) proposes clearly defined normal recovery trajectories (differing with both admission and operation type) in 210 patients following both minimally invasive and open abdominal and thoracic surgery [40]. Through wearable monitoring postoperative recovery “trajectories” can be quantified and continuously tracked to inform timely intervention and counselling to improve postoperative mobility.

However, most studies tended to employ a “snapshot” capture of activity levels over a set time (for instance 24 h or 7 days) preoperatively which was compared to similar postoperative data capture after a set recovery duration (for instance 6 months) [32, 37]. A limitation of this “snapshot” approach is the lack of continuous data capture over the duration of postoperative recovery which may not reflect how recovery outcomes may improve and decrease over a unique recovery trajectory [40]. Such snapshots, for example Thewlis et al.’s (2019) report of no significant difference between preoperative and postoperative activity profiles in terms of sedentary duration (620 ± 143 min/day preoperatively versus 641 ± 133 min/day, respectively) may not reflect fluctuations over the course of recovery [37]. Additionally, arbitrary study period of 3 months or 6 months may not necessarily be sufficient duration for these recovery trajectories that differ with operation type, admission and patient characteristics [40]. As such, Matsunaga-Myoji et al. (2020) reports improving (moderate-vigorous) physical activity levels (58.3 versus 72.3 min/week, *p* = 0.008) between 1 and 3 years postoperatively, following total knee replacement. [35]

Findings from the few studies which have undertaken continuous and objective activity tracking, for example by Steinen et al. (2020) and Scheer et al. (2017), suggest patient recovery following spinal surgeries may also follow these defined trajectories [21, 22]. A challenge facing continuous recovery trajectory monitoring remains patient compliance, with only 68% of Carmichael et al.’s participants completing follow-up at four weeks postoperatively [40]. These issues are not consistent, with some included studies also reporting follow-up data for > 90% of recruited participants [20, 29, 30]. Future studies may explore methods of participant retainment and compliance, such as incentives, gamification and/or counselling.

The continuous stream of objective data regarding patient performance provided by wearable monitoring may also be used to predict recovery outcomes. Regression analysis by Taniguchi et al. (2016) demonstrated postoperative physical activity in the first month predicted activity levels up to 6 months postoperatively (following total knee arthroplasty) to be predicted [30]. Pre-operative mobility characteristics were used for similar predictive modelling of recovery outcomes following total hip and knee arthroplasty by Lebleu et al. (2021) [36]. Existing risk-prediction models based on patient-reported and/or functional outcome measures [41], may benefit from such objective data capture from wearable monitoring.

## Strengths and limitations

To our knowledge, this is the first review of wearable monitoring (of postoperative recovery) following hip, knee, and spine surgery. Other strengths include systematic search of literature from date of inception to February 28^th^, 2021, across 3 unique databases as well as standardised quality assessments of included studies via the Newcastle Ottawa Scale. However, limitations include restriction of search to only hip, knee, and spine surgeries – despite these encompassing the majority. Future studies may explore surgery for other gait altering pathologies – such as deep-brain stimulation for Parkinson’s, vascular claudication as well as ankle surgery.

## Conclusion

Elective orthopaedic procedures are likely a very suitable patient population to benefit from wearable monitoring with their recovery and rehabilitation directly related to their walking health and mobility. Wearable monitoring may also enable timely postoperative care and intervention during recovery providing benefits to patients, healthcare providers and insurance providers alike since orthopaedic surgeries comprise a significant proportion of health care. Predictive modelling of post-recovery outcomes, and development of recovery trajectories from common orthopaedic procedures may enable timely mobility interventions to assist postoperative rehabilitation.

## Data Availability

The datasets used and/or analysed during the current study are available from the corresponding author on reasonable request.
